# RETRACTED ARTICLE: Genotoxicity and alteration of the Gene Regulatory Network expression during *Paracentrotus lividus* development in the presence of carbon nanoparticles

**DOI:** 10.1007/s43188-020-00081-y

**Published:** 2021-04-12

**Authors:** Elisabetta Carata, Bernardetta Anna Tenuzzo, Stefania Mariano, Andrea Setini, Marco Fidaleo, Luciana Dini

**Affiliations:** 1 Department of Biological and Environmental Science and Technology, Di.S.Te.B.A. University of Salento, Lecce, Italy; 2 Department of Biology and Biotechnology “C. Darwin”, Sapienza University of Rome, Rome, Italy; 3 CNR-Nanotec, Lecce, Italy

The Editor-in-Chief has retracted this article. After publication, two of the panels in Figure 1C were found to overlap with figures in another published article [1], specifically: Figure 1C left panel overlaps with Figure 2A of [1]. Figure 1C middle panel overlaps with Figure 6 of [1]. The Editor-in-Chief therefore no longer has confidence in the integrity of the data in this article. The authors have agreed to this retraction but not to the wording of this retraction notice. [1] Manno D, Serra A, Buccolieri A, Panzarini E, Carata E, Tenuzzo B, Izzo D, Vergallo C, Rossi M, Dini L (2013) Silver and carbon nanoparticles toxicity in sea urchin Paracentrotus lividus embryos. BioNanoMat 14(3–4):229–238.

## Supplementary Information

Below is the link to the electronic supplementary material.

Former article version (PDF 1612 KB)

