# Supplementary material for: RETRACTED ARTICLE: Genotoxicity and alteration of the Gene Regulatory Network expression during Paracentrotus lividus development in the presence of carbon nanoparticles
Source: Toxicol Res. 2021 Apr 12;38(2):257. doi: 10.1007/s43188-020-00081-y (PMC8960529; doi:10.1007/s43188-020-00081-y)
Supplement: Supplementary file 1 — Former article version (PDF 1612 KB) [file 43188_2020_81_MOESM1_ESM.pdf]

Toxicol Res.
https://doi.org/10.1007/s43188-020-00081-y

eISSN 2234-2753
pISSN 1976-8257

Toxicological Research

ORIGINAL ARTICLE

Check for updates

Genotoxicity and alteration of the Gene Regulatory Network expression during *Paracentrotus lividus* development in the presence of carbon nanoparticles

Elisabetta Carata[1] · Bernardetta Anna Tenuzzo[1] · Stefania Mariano[1] · Andrea Setini[2] · Mauro Fidaleo[2] · Luciana Dini[2,3]

Received: 1 September 2020 / Revised: 13 November 2020 / Accepted: 17 December 2020
© The Author(s) 2021

RETRACTED ARTICLE

Abstract

Nanoparticles are a newly emerging class of pollutants with eco-toxicological impacts on marine ecosystems; they are characterized by nano-scale size which improves their physical, chemical and biological properties. To better understand the mechanisms of embryotoxicity of carbon-based nanoparticles, the genotoxicity and the perturbation of the Gene Regulatory Network (GRN) expression have been investigated during the development of the sea urchin *Paracentrotus lividus* from fertilization to early pluteus stage. Increasing quantities of carbon nanoparticles (C-NPs), 0.5, 2.5 and 25×10^{13} C-NPs/500 cm^3 of filtered seawater were administered during fertilization and the development was monitored up to the early pluteus stage (48 h). DNA damage and gene expression were assayed by Comet assay and Real-Time PCR, respectively. Taken together, our results indicate that embryo malformations taking place in the presence of C-NPs are due to altered regulation of the GNR and to a progressive accumulation of DNA single-strand breaks.

Keywords Embryotoxicity · Genotoxicity · Gene regulatory network · Sea urchin · Carbon nanoparticles

Introduction

The aquatic environment is under the pressure given by the significant increase of the use of nanomaterials (NMs) in consumer and industrial products, whose potential biological effects is still under debate [1]. The rising global concern about NMs toxicity is mainly focused to clarify the possible risk of engineered nanoparticles (E-NPs), such as metals, metal oxides, carbon nanotubes, fullerenes, polymeric E-NPs, quantum dots, etc. that intentionally or accidentally can be released into the soil, water and sea [2]. E-NPs are emerging contaminants and many literature data support type- and size-related E-NPs toxicity in aquatic organisms [3]. However, since E-NPs are generally found in the aquatic environment at very low concentrations, it is likely that they exert sub-lethal effects on the organisms [4]. The majority of the studies have focused to identify the E-NPs toxicity by analyzing many different parameters, e.g., mortality, developmental defects, and, more recently, genotoxic potential, like DNA or/and chromosomal fragmentation, or DNA strand breaks, but at unrealistic high concentrations [4, 5]. The number of papers dealing with the E-NPs toxicity is very consistent and continuously increasing; moreover papers about E-NPs genotoxicity are fasting growing. However, a gap of knowledge regarding the underlying mechanisms of E-NPs toxicity and/or genotoxicity and/or modulation of gene expression still exists. With the aim to fill this gap, there is increasing attention to the possible E-NPs role on the gene expression, which, in turn, could help to clarify the involvement of E-NPs action mechanisms in the sub-lethal effects.

Among E-NPs, carbon nanoparticles (C-NPs) are currently considered key elements in nanotechnology for their versatility. Their potential applications range from biomedicine through nanoelectronics to mechanical engineering and to know their exposure-related hazards is pivotal. Due to the

✉ Luciana Dini
luciana.dini@uniroma1.it

1 Department of Biological and Environmental Science and Technology, Di.S.Te.B.A. University of Salento, Lecce, Italy

2 Department of Biology and Biotechnology "C. Darwin", Sapienza University of Rome, Rome, Italy

3 CNR-Nanotec, Lecce, Italy

hydrophobic nature and the high surface-adsorption potential [6, 7], C-NPs may be a serious risk for biota since they can attach to different surfaces, including organisms, [7, 8]. Although the numerous publications about the E-NPs impact on the marine environment [7, 9–22], there is still a lack of knowledge in this area.

In our previous studies, we already reported data on the C-NPs induced morphological alterations, on the C-NPs stress response impairment, and on biomineralization process of sea urchin embryos [18, 23, 24]. Here, we investigated the molecular mechanisms leading to morphological modification. Sea urchin morphology is under the Gene Regulatory Network (GRN) control that specifies the main ectodermal territories (ventral ectoderm, dorsal ectoderm and ciliary band) and the molecular mechanisms involved in regionalization of the embryo along the D/V axis [25, 26]. Saudemont et al. identified 21 genes encoding for transcription factors and signalling molecules involved in sea urchin embryo development (goosecoid, nk2/2, tbx2/3, nk1, foxA, brachyury, foxG, onecut/hnf6, irxA, hox7, dlx, smad6, msx, id, oasis, deadringer, otx, gfi, pax2/5/8, atbf1, unc4), 13 signalling molecules (nodal, lefty, bmp2/4, chordin, fgfA, fgfr1, glypican 5, admp2, bmp1, wnt8, univin, wnt5, delta), 2 RNA binding protein (rkhd, ptb), 3 differentiation genes (*cyIIIa, 29D, tubulinβ3*) and 1 mitochondrial gene (*cytochrome oxidase*) [25, 26].

In this paper, we investigated the genotoxic risks and the effects on the ectodermal (GRN) expression of different C-NPs quantities exposure, during the development of *Paracentrotus lividus* from fertilization to early pluteus.

Materials and methods

Synthesis and characterization of C-NPs

C-NPs were obtained as reported in [24]. Briefly, high purity graphite rod (99.99%, [illegible] Supplies Division Structure Probe Inc., West Chester, PA, USA) was used as an anode (5 mm diameter), and a stainless-steel rod AISI 1016, 5 mm diameter (Swagelok, Solon, OH, USA) was used as a cathode. The electrodes were immersed in 100 ml of deionized water (MilliQ 18.2 [illegible] Millipore, Merck KGaA, Darmstadt, Germany) at a reciprocal distance of 10 mm. In the electrolysis process, the electric power applied to the electrodes was at a constant voltage of 30 V. Simultaneously, the colloidal solution was forcedly dispersed by an ultrasonicator, which was the flat-type ultrasonic equipment (Flexonic-1200-35/72/ 100G, Mirae Ultrasonic Tech., 402-1101 Bucheon Techno Park 193 Yakdae-dong, Zip: 421808, Bucheon Si, Gyeonggi, Korea). To prevent the aggregation of NPs, the ultrasonicator was continuously operated at the power output of 1000 W with a frequency of 100 ± 5 kHz during the production of NPs. At the end of the reaction, the solution of NPs is diluted with distilled water (20 ml solution of NPs and 30 ml of water). Characterization of C-NPs in MilliQ and in filtered sea water (FSW) in terms of size distribution, polydispersity index (PDI) and ξ-potential, as well as measurement of conductance of the surrounding media, were determined using a ξ-potential and particle size analyser Brookhaven ZetaPlus (Brookhaven Instruments Corp., Holtsville, NY, USA), at 25 °C and angle scattering of 90°.

Gametes quality

Adult *P. lividus* sea urchins were collected along the Salento Ionian coasts, Apulia, Italy. Animals were induced to shed gametes by intracoelomic injection of 0.5 M KCl. Gametes were evaluated for their optimal maturation before fertilization. The percentage of mature oocytes released from the gonads was calculated and the solutions with a percentage of immature oocytes higher than 10% discharged. The spermatozoa quality, in terms of motility and fertilization rate, was carried out by the exposition of 10 µl of sperm suspensions to 0.5, 2.5 and 25 × [illegible]-NPs/500 cm^3 of FSW. Negative and positive controls were performed by incubating spermatozoa (a) diluted in FSW; (b) diluted in $K_2Cr_2O_7$ in accordance with the Italian guidelines for *P. lividus* [27–29].

Embryos culture and C-NPs treatments

Eggs, washed several times with FSW, were fertilized in flasks containing 200 ml of FSW at a rate of 40 eggs/ml with 100 µl of sperms suspension diluted 1:10,000. Sperms were added simultaneously with the different quantities of C-NPs in FSW. Fertilized eggs were cultured under aseptic conditions at 16–18 °C, with a dark/light period and with controlled oxygen flow in Millipore FSW containing antibiotics up to early pluteus stage (48 h from fertilization) for gene expression and DNA damage and up to 72 h for the morphological analysis.

The *P. lividus* embryos were divided in four different groups: (1) normal control without C-NPs; (2) 0.5×10^{13} C-NPs in 500 cm^3; (3) 2.5×10^{13} C-NPs in 500 cm^3; (4) 25×10^{13} C-NPs in 500 cm^3. At 30 min, 6, 18, 24, 48 and 72 h from sperm and C-NPs addition, the embryos were collected by low-speed centrifugation and processed for the specific analysis as reported below.

Assessment of morphological changes, DNA damage and gene expression

The development was monitored by morphological analysis of control and C-NPs treated embryos (50 individuals per condition) using an inverted microscope Nikon Eclipse 80i (Nikon, Nikon Instruments Europe, Amstelveen,

RETRACTED ARTICLE

Netherlands) and images were taken by a digital camera Nikon DMX 1200F.

DNA damage was assessed by Comet assay, performed according to [30], with some modifications. Briefly, 60 µL of exposed eggs suspension from at least three specimens of *P. lividus* was mixed with 100 µL of 0.7% low-melting point agarose in a salt solution (Buffer A) (0.4 M NaCl, 9 mM KCl, 0.7mMK_2PO_4, 2 mM $NaHCO_3$). 70 µL of the latter suspension were spread in duplicate onto microscope slides, coated with 150 µL of 1% normal melting point agarose in Buffer A and with a cover slide. After the agarose had solidified, the cover was removed and the slide was immersed for 1 h in a cold lysing solution (2.5 M NaCl, 10 mM TrisHCl, 100 mM ethylendiaminetetracetic acid (EDTA), 1% sodium N-lauroyl sarcosinate, 1% Triton X-100 and 10% dimethyl sulfoxide (DMSO), pH 10) at 4 °C in the dark. After two rinses in distilled water, slides were incubated in Alkaline Buffer (300 mM NaOH and 1 mM EDTA, pH 13) for 25 min at 4 °C in the dark. Then, electrophoresis was performed for 20 min at 25 V, 330 mA in Alkaline Buffer. The slides were neutralized in 0.4 M TrisHCl (pH 7.4), dehydrated in absolute ethanol for 5 min and air-dried before storage at 4 °C until use. Slides were stained with ethidium bromide (20 µg/ml) and analysed by Leica DM750 Fluorescence Microscope (Leica Microsystems GmbH, Wetzlar, Germany). Images were taken with a CCD Leica ICC50 HD camera coupled to an epifluorescence microscope and analysed with Comet Score TM (freeware from TriTek Corp., Summerduck, VA, USA). The tail moment was used as a measure of DNA strand breaks. For each sample, three slides were scored by counting fifty-seventy nuclei per slide [31].

The gene expression was evaluated by Real-Time PCR. Total RNA was isolated according to the manufacturer's instructions using kit PureLink ™ RNA Mini Kit (Invitrogen, Carlsbad, CA, USA) from collected embryos, frozen in liquid nitrogen and stored at −80 °C until use. The quantitative and qualitative analysis of the extracted RNA was performed by electrophoresis on [illegible]% agarose gel, under denaturing conditions (formamide 50%, 3-[*N*-morpholino] propanesulfonic acid [illegible] [MOPS], formaldeide 5.5%) and quantified by a spectrophotometer (Thermo Scientific GENESYS spectrophotometer, Waltham, MA, USA). Complementary DNAs (cDNAs) were synthesized according to the manufacturer's suggestions of single-step ThermoScript™ RT-PCR System kit (Invitrogen, Carlsbad, CA, USA) protocol. Table S1 in supplementary materials lists genes and time-points after fertilization that were taken into account.

In order to quantify the gene expression, 1% of each retrotrascription reaction was used to carry out Real-Time PCR on a SmartCycler System (Cepheid, Sunnyvale, CA, USA) with SYBR® Green JumpStart TaqReadymix (Sigma-Aldrich, St. Louis, MO, USA). The primer pairs are indicated in Table S1. Real-Time reaction was performed in triplicate for each sample. Quantification of relative gene expression was performed as described by the manual of Applied Biosystems Step One Plus real time PCR, a *Comparative Threshold Cycle Method*, using SYBR Green chemistry [32]. The Real-Time PCR reaction was performed after initial denaturation for 2 min at 94 °C, followed by 35 cycles of 94 °C, 15 s; 55 °C, 30 s; 72 °C, 30 s. The Pl-S24 gene [33] that encodes for a ribosomal protein constitutively expressed was considered as reference gene. The relative quantitative analysis of the amplicons was performed using method "$2^{-\Delta\Delta Ct}$" (cycle threshold), which provides a data normalization of the treated samples compared to housekeeping gene.

Statistical analysis

All the experiments were carried out by independent triplicates. Statistical analyses were carried out with the software Past, version 4.03. For morphological analysis one-way analysis of variance (ANOVA) was performed, while the classification of means was done using the least significant difference (Student's t test) at $p < 0.05$. For Comet assay analysis, Kruskal–Wallis and one-way analysis of variance (ANOVA) was performed, while the classification of means was done using the least significant difference (Duncan's test) at $p < 0.05$.

Results

CNPs characterization

The C-NPs were extensively characterized, with a particular focus on the C-NPs clusters/aggregates formation in FSW (37‰ salinity). Shape, size, size distribution and crystallinity were assayed with TEM (Fig. 1). C-NPs size and polydispersity index (PDI) was further analysed with DLS. Freshly prepared C-NPs in MilliQ water were round and well dispersed; nanoparticles aggregation slightly increased with time and increasing C-NPs quantities. In FSW, C-NPs showed an increased particles size, slowly increased with time (from about 20 nm to about 60 nm of diameter), without shape changes and with ξ-potential always negative (Fig. 1).

C-NPs interfere with the development of *P. lividus*

Only the eggs showing the vitelline envelope, considered zygotes, were counted. The rate of fertilized eggs was 96 ± 3% in controls and decreased by about 20% in the treatments (Fig. 2). The appearance of the vitelline envelope in the presence of C-NPs was delayed of about 20 min compared to controls.

RETRACTED ARTICLE

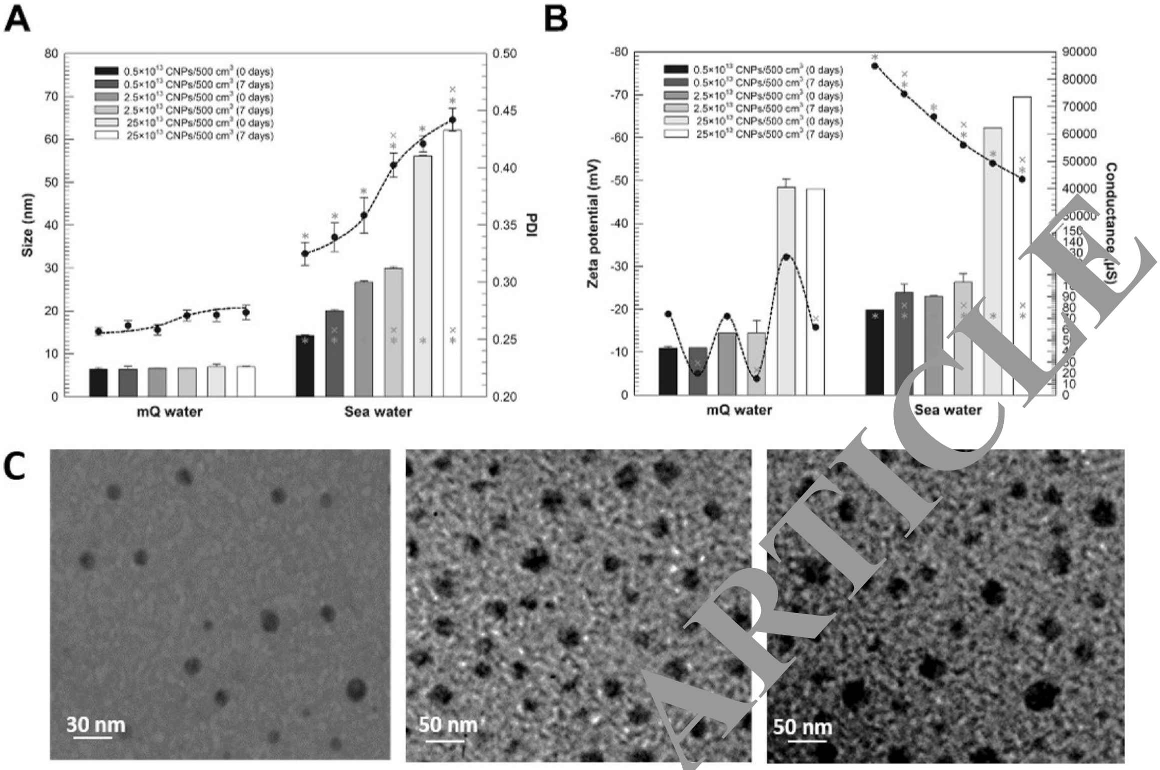

Fig. 1 Size distribution and polydispersity index (PDI) of C-NPs d[illegible]persed in MilliQ water and FSW. At least three independent experi[illegible] ments with two replicates were performed by using automa[illegible]ic optimization of analytical conditions and processed by th[illegible]software ZetaPals Particle Sizing ver. 3.86 (Brookhaven Instrum[illegible]Corp., [illegible]tsville, NY, USA). The difference among the independent experime[illegible]ts, measured as SEs, never exceeded 1%. TEM micrographs of [illegible]-NPs. **a** Freshly prepared C-NPs are round and well dispersed with a size distribution ranging from 2 to 12 nm and an average diameter of 7 ± 2 nm. **b**, **c** C-NPs in FSW show an increasing of size

The embryotoxicity of the three diffe[illegible]ent C-[illegible] quantities to *P. lividus* is not C-NPs con[illegible]entration-dependent. The modifications observed during [illegible]2 h of development are summarised in Fig. 3, where the n[illegible], modified and dead embryos percentages from [illegible]ization to pluteus stage are reported. The category of m[illegible]ed embryos includes embryos with asynchron[illegible]s clea[illegible]age, with asymmetric cleavage, with acceler[illegible]d [illegible]elerated cleavage rate. In Fig. 3 representat[illegible]on of t[illegible] main morphological modifications are report[illegible].

At the ea[illegible]liest stag[illegible] of development, the most frequent malform[illegible]tions were asynchronous divisions and delayed vitelline [illegible]elop[illegible] formation (after 30 min instead of 5/1[illegible]n fro[illegible]e addition of sperms) causing embryos to s[illegible] to[illegible]bottom of the flask. At later stages of developmen[illegible]modifications are mainly related to arms and spicules formati[illegible]n.

As mentioned above, development modifications are indirectly related to the C-NPs quantities. The highest C-NPs quantity showed mild or null influence on the development until pluteus stage, while the lowest C-NPs quantity was the most embryotoxic, in which asymmetric arms, swollen gut, and C-NPs accumulation in the gut were observed (Fig. 3).

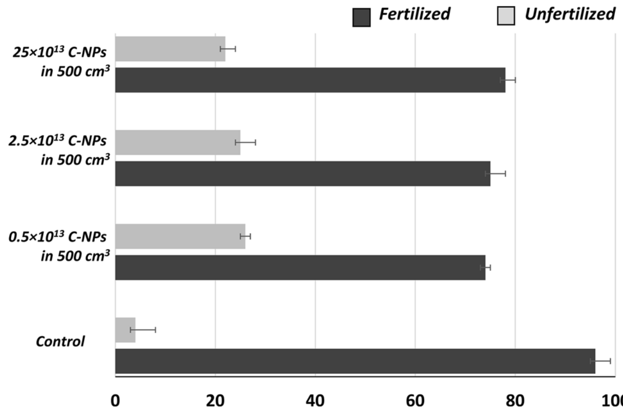

Fig. 2 Rate of fertilized and unfertilized eggs of sea urchin *P. lividus* embryos at 30 min after sperms and C-NPs addition. The difference among the independent experiments, measured as SEs, never exceeded 1%

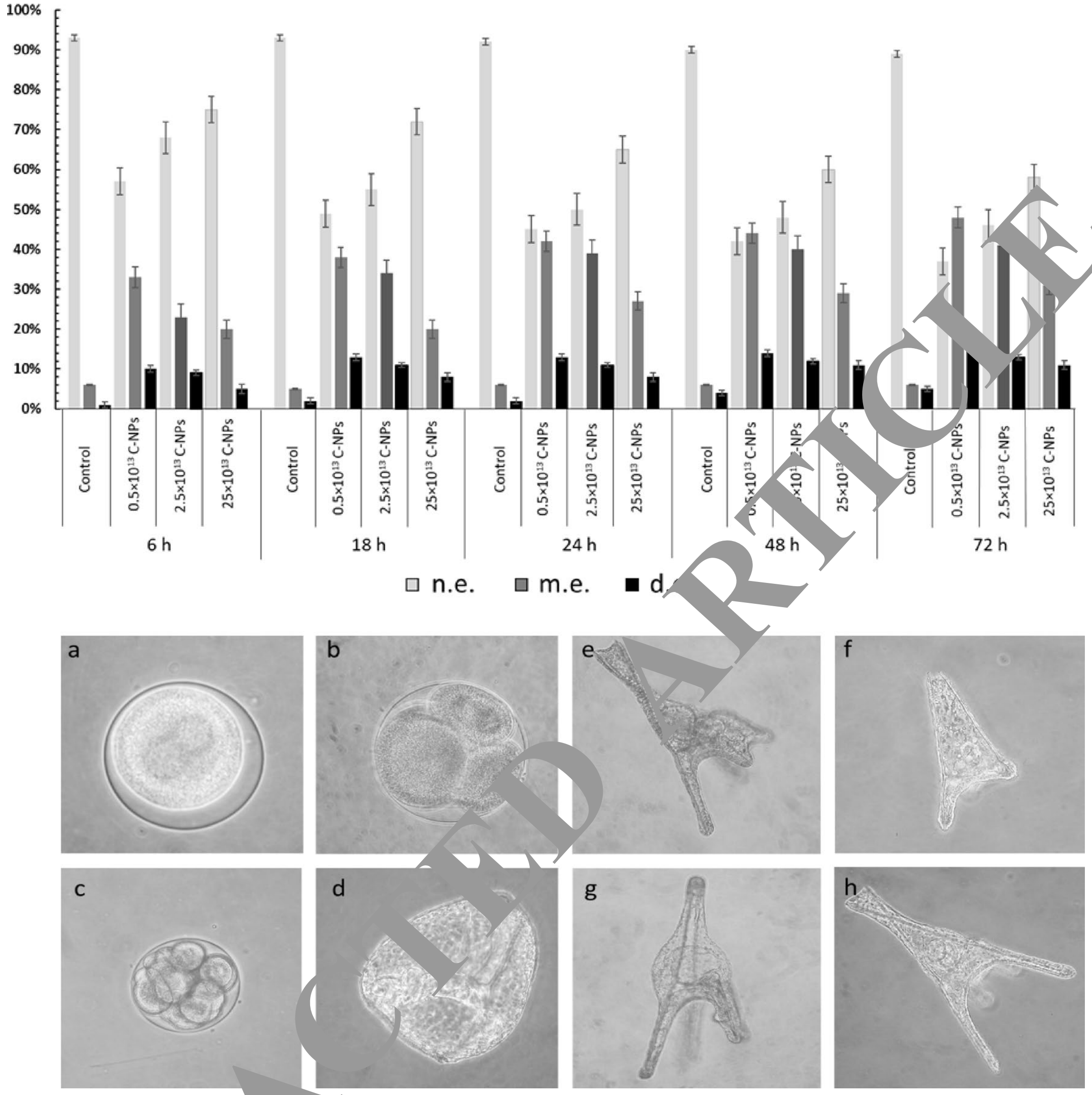

Fig. 3 Rate of normal (n.e.) [illegible]ified (m.e.) dead (d.e.) sea urchin *P. lividus* embryos incubated with different amounts of C-NPs. The difference among the independent experiments, measured as SEs, never exceeded 1%. In the figure are reported the representative images of the main alterations observed during the development of sea urchin exposed to different concentrations of nanoparticles. (Nikon Eclipse 80i) **a** perturbation of the lifting of the fertilization envelope; **b** asynchronous segmentation; **c** asymmetric blastomeres; **d** abnormal gastrula; **e** pluteus with asymmetric arms and altered spicules; **f** early pluteus with undeveloped arms; **g** pluteus with asymmetric arms and swollen gut; **h** pluteus with altered formation of spicules

RETRACTED ARTICLE

C-NPs induce DNA single-strand breaks

Comet [illegible] revealed DNA damages in eggs incubated with different quantities of C-NPs (Fig. 4). The highest DNA damage index, expressed as Tail Moment, was observed in the presence of 2.5×10^{13} C-NPs/500 cm^3 C-NPs, that corresponded to the intermediate quantity used in our experiments. The DNA damage was 2.3 times more the DNA damage of the negative control, and 1.53 times more the positive control in presence of 2.5×10^{13} C-NPs/500 cm^3 AgNPs.

C-NPs interfere with ectodermal gene regulatory network

One of the possible mechanisms of action underlying the embryotoxicity of C-NPs to *P. lividus* could be found in the gene expression modulation of the embryo regionalization along the D/V axis (GRN). Among the GRN, *nodal, lefty, bmp2/4, tbx2/3, wnt5, wnt8* and *univin* genes were selected; other investigated genes were those controlling the skeletogenesis, *msp130*, *SM50, SM30* and *PM27* genes. In Table 1 is reported the list of genes and the respective

A

B

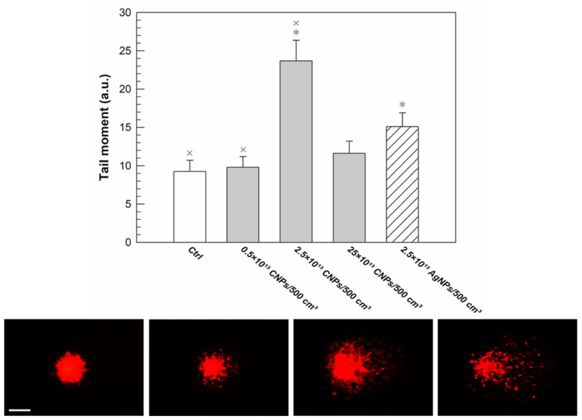

Fig. 4 Analysis of DNA damages of eggs treated for 6 h with C-NPs with 0.5×10^{13} C-NPs in 500 cm^3, 2.5×10^{13} C-NPs in 500 cm^3, 25×10^{13} C-NPs in 500 cm^3; in the positive control the eggs exposed to 2.5×10^{13} AgNPs in 500 cm^3. **A** Comet Assay data quantitation by specialized CA software CometScore—a graph representing the Tail Moment (TM). This parameter essentially represents the product of the percentage of total DNA in the tail and the distance between the centres of the mass of head and tail regions. **B** Representative Comet images, from null DNA damage (a) to maximum DNA damage (b), of eggs stained with ethidium bromide and observed under fluorescent microscope (Leica DM750 Fluorescence Microscope)

analysis during the development of *P. lividus* sea urchin embryos as suggested by [25, 34–37].

GRN expression

All the genes examined showed significant gene expression alterations. After 6 h from fertilization, corresponding to early blastula stage, *nodal, lefty* and *bmp2/4* gene expression has been influenced by the C-NPs presence and partially by the C-NPs quantities (Fig. 5a); the lower and intermediate quantities of C-NPs induced overexpression of the genes, fold changes from 1.72 to 4.81 (Fig. 5a). Interestingly, the highest quantity of C-NPs did not cause significant alteration in the expression (fold changes from – 0.32 to 1.07) of *nodal* and l*efty*, while *bmp2/4* is weakly over-expressed (fold change 3.76) (Fig. 5a).

After 18 h after fertilization, corresponding to the blastula stage, *tbx2/3* and *wnt5* gene expression were strongly downregulated in embryos treated with the highest and intermediate C-NPs quantities, while a moderate downregulation for tbx2/3 and null regulation for *wnt5* was measured with the lowest C-NPs quantity (Fig. 5b). The *wnt8* gene expression, which is closely related to *nodal* through a secondary positive feedback mechanism, was slightly downregulated with the intermediate and the highest C-NPs quantities (Fig. 5b) (fold change – 0.69 and – 0.75). The lowest C-NPs quantity determined no expression changes (fold change 0.2).

After 24 h from fertilization, corresponding to the gastrula stage, the gene expression *of univin*, which belongs to transforming growth factor-beta (TGFβ) family and is a key regulator of ectodermal patterning, was upregulated in all treatment (fold change 3.[illegible], 3.1 and 4.2) (Fig. 5c).

After 4[illegible] h from fertilization, corresponding to early pluteus stage, skeletogenesis gene expression of *msp130* gene was upregulated only with the highest C-NPs quantity (fold change 1.7), and practically unchanged with low and medium C-NPs quantities (Fig. 5d). The *SM50, SM30* and *PM27* genes expression was considerably upregulated with all the C-NPs quantities (Fig. 5d). The most efficacious inducer of gene expression was the lower C-NPs quantity.

Discussion

The results of the present investigation are in line with the studies reporting on the eco-toxicological impacts on marine ecosystems of E-NPs [1]. Studies on invertebrates and fishes have already suggested harmful effects of E-NPs exposure [20, 38, 39]. Our contribution to this area is given by pieces of evidence of the altered GRN expression and genotoxicity of C-NPs exposure in *P. lividus* embryos. Carbon-based NMs are an attractive family of NMs which comprises

Table 1 List of genes and respective analysis during development of *P. lividus* sea urchin embryos

Gene	Developmental time and stage	Origin	References
nodal	6 h/early blastulae	Zygotic	Saudemont et al. [25]; Duboc et al. [35]
Bmp2/4	6 h/early blastulae	Zygotic	Saudemont et al. [25]; Duboc et al. [34]
lefty	6 h/early blastulae	Zygotic	Saudemont et al. [25]; Duboc et al. [35]
wnt5	18 h/blasulae	Zygotic	Saudemont et al. [25]; McIntyre et al. [36]
wnt8	18 h/blastulae	Zygotic	Saudemont et al. [25]
tbx2/3	18 h/blastulae	Zygotic	Saudemont et al. [25]
univin	24 h/gastrulae	Zygotic	Saudemont et al. [25]; Shashikant et al. [37]
msp130	48 h/early pluteus	Zygotic	Shashikant et al. [37]
SM50	48 h/early pluteus	Zygotic	Shashikant et al. [37]
SM30	48 h/early pluteus	Zygotic	Shashikant et al. [37]
PM27	48 h/early pluteus	Zygotic	Shashikant et al. [37]

RETRACTED ARTICLE

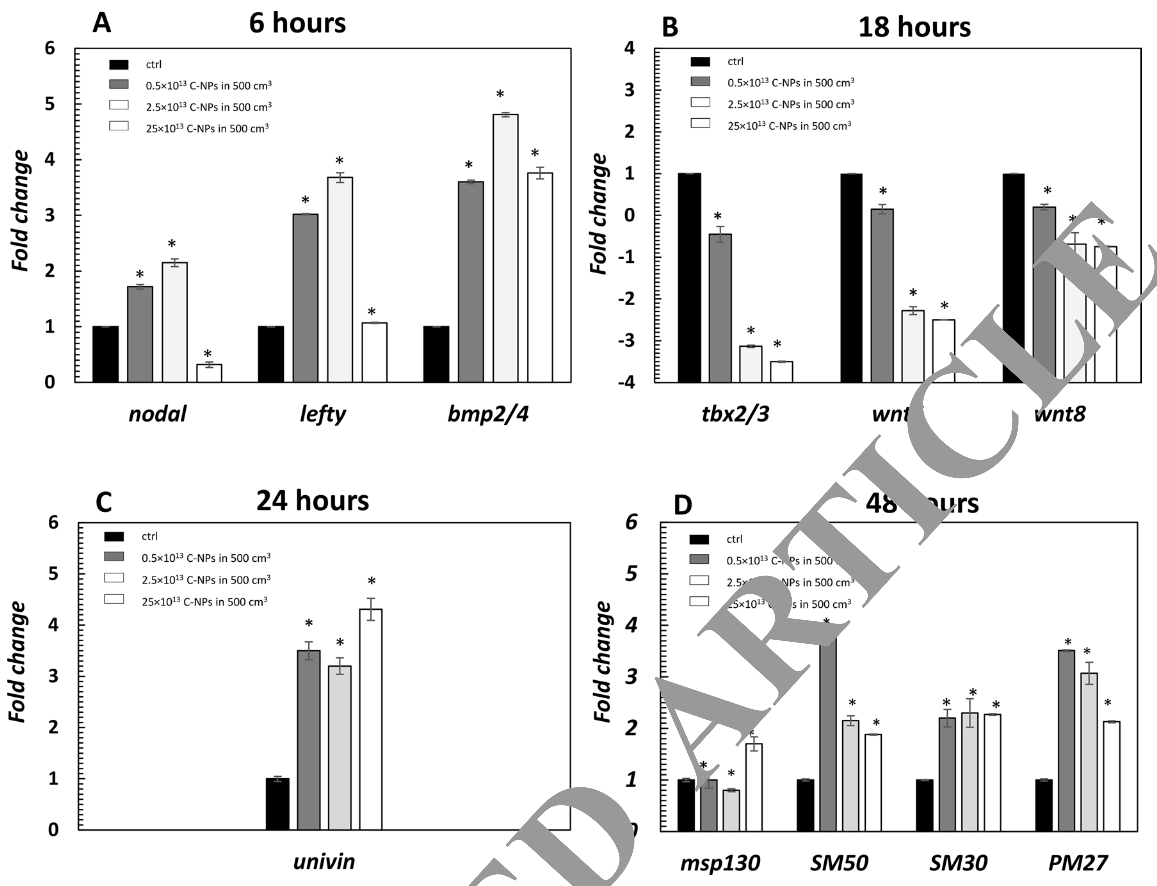

Fig. 5 Alteration of gene expression analyzed in embryos [illegible] *livi-dus* treated with different amounts of C-NPs. **a** *nodal* [illegible] 6 h after fertilization, fold change of 1.72 with [illegible] C-NPs in 500 cm^3; 2.15 with 2.5×10^{13} C-NPs in [illegible] with 25×10^{13} C-NPs in 500 cm^3; *lefty* gene exp[illegible]ession 6 h a[illegible]er fertilization, fold change of 3.02 with 0.5×10^{13} C-NPs in 500 cm^3, 3.68 with 2.5×10^{13} C-NPs in 500 cm^3, 1.07 [illegible] C-NPs in 500 cm^3; *bmp 2/4* gene expression 6 h [illegible]ertilization, fold change of 3.6 with 0.5×10^{13} [illegible] in 500 cm^3, 4.81 with 2.5×10^{13} C-NPs in 500 cm^3, 3.76 [illegible]$\times10^{13}$ C-NPs in 500 cm^3. **b** *tbx2/3* gene expression 18 h [illegible]ter fertilization fold change of −0.45 with 1×108C-NPs, [illegible]3.13 [illegible] 2.5×10^{13} C-NPs in 500 cm^3, −3.5 with [illegible] cm^3; *wnt5* gene expression 18 h after fertilization, fold [illegible]nge of 0.15 with 0.5×10^{13} C-NPs in 500 cm^3, −2.28 [illegible] C-NPs in 500 cm^3, −2.5 with 25×10^{13} C-NPs [illegible]m^3; *wnt8* gene expression 18 h after fertilization, fold c[illegible]nge of [illegible] 0.5×10^{13} C-NPs in 500 cm^3, −0.69 with [illegible] C-NPs in 500 cm^3, −0.75 with 25×10^{13} C-NPs in 500 cm^3. **c** *univin* gene expression 24 h after fertilization, fold change of 3.5 with 0.5×10^{13} C-NPs in 500 cm^3, 3.2 with 2.5×10^{13} C-NPs in 500 cm^3, 4.31 with 25×10^{13} C-NPs in 500 cm^3. **d** *msp130* skeleton gene expression 48 h after fertilization, fold change of 1.01 with 0.5×10^{13} C-NPs in 500 cm^3, 0.8 with 2.5×10^{13} C-NPs in 500 cm^3, 1.70 with 25×10^{13} C-NPs in 500 cm^3; *SM50* gene expression 48 h after fertilization, fold change of 3.76 with 0.5×10^{13} C-NPs in 500 cm^3, 2.15 with 2.5×10^{13} C-NPs in 500 cm^3, 1.88 with 25×10^{13} C-NPs in 500 cm^3, *SM30* gene expression 48 h after fertilization, fold change of 2.2 with 0.5×10^{13} C-NPs in 500 cm^3, 2.3 with 2.5×10^{13} C-NPs in 500 cm^3, 2.27 with 25×10^{13} C-NPs in 500 cm^3; *PM27* gene expression 48 h after fertilization, fold change of 3.51 with 0.5×10^{13} C-NPs in 500 cm^3, 3.07 with 2.5×10^{13} C-NPs in 500 cm^3, 2.13 with 25×10^{13} C-NPs in 500 cm^3. Each bar represents the mean of three independent experiments ± SD. Asterisks indicate significant differences from respective control values at the same concentration ($p < 0.05$) according to the one-way ANOVA ($p < 0.05$)

[illegible]bon allotropic forms as amorphous carbon, grap[illegible]e and nanodiamonds, carbon nanotubes and carbon nanodo[illegible]s. Nano-diamonds and graphene oxides at concentration of 5000 μg/ml decrease basic fibroblast growth factor (bFGF) mRNA expression and protein level in the heart of chicken embryos [40, 41]. In mice embryos, skeletal malformations were described following intraperitoneal injection of multiwall carbon nanotubes [42]. In previous works on *P. lividus*, we reported that C-NPs induce: (1) embryotoxicity, leading to abundant malformed embryos formation and an acceleration of developmental stages; (2) alteration of the *14-3-3* epsilon mRNA levels with a consequent stress response; (3) alteration of the *Sp-CyP-1* m-RNA levels with consequent induction of biomineralization process in the spicules [17, 18, 23, 24]. In the present work, our results show that C-NPs embryotoxicity is not directly dependent on the C-NPs quantities. As the C-NPs quantity increased, the toxicity became milder. This is in line with the toxicity

degree that is reported to be inversely related to the size of the NPs, i.e. the bigger is the particle size the lower is toxicity (Fig. 3) [43]. However, the shape of the NPs is also linked to the severity of toxicity [44]. The toxicity here reported is most likely dependent on the size of the C-NPs but not on the shape. Indeed, C-NPs in the FSW underwent to size increase and maintained an unmodified round shape. In our system, the decreased toxicity was due to the increased size and not to the formation of C-NPs agglomerates; in accord, polydispersity index (PDI) values are in line with a good polydispersity. The good polydispersity of C-NPs favours internalization and ingestion of C-NPs. We already reported that plutei ingested C-NPs despite their slightly increased size in FSW, and once inside the gut, C-NPs were modified into aragonite with a high bio-mineralization order [23].

Malformations and alteration of the normal development patterning caused embryos' deaths. The latter is the expression of altered molecular mechanisms and possible DNA damages.

Genotoxic responses, i.e. DNA strand breaks and chromosomal fragmentation, and oxidative stress in *Arbacia lixula* embryos exposed to CuO NPs were reported [4, 10]. We found that C-NPs were genotoxic to *P. lividus* embryos as detected by comet assay, which is generally the preferred method due to its capacity to detect low levels of DNA damage as reported by comparing eight different genotoxicity assays [30, 45].

DNA damages significantly increase in the presence of intermediate C-NPs quantities. This has induced impairment of division and transcription that explains the modification during development. Malformations of embryos are the result of the modulation of genes involved in development. During sea urchin development, the ectoderm is the source of signals that pattern all three germ layers along the dorsal–ventral (D/V) axis. The patterning of all three germ layers relies on the activity of signalling centre located in the ventral ectoderm necessary to define dorsal–ventral polarity of embryo. Studies on the genes that regulate ectoderm patterning and morphogenesis of the embryo along the D/V axis, led to the identification of *TGF-β, nodal, bmp2/4* and *univin* as key regulators of ectoderm patterning in *P. lividus* [34, 46–48].

It is also reasonable that specific substance can activate a late gene in an early stage of development (or vice versa, a substance can induce/keep inducing an early gene in a late phase) thus leading to a malformation or death. In the present work, we evaluated alterations of GRN detecting the expression of genes at the expected time points and validating gene alterations by morphological observations.

During the development, the specification of cell fate is regulated by *nodal*, which is responsible of the dorsal–ventral axis determination and encodes for a protein belonging to the TGF-β superfamily [49]. Modulation of *nodal* signalling is essential for normal patterning of D/V axis that is modulated by its antagonist, *lefty*. The *TGF-β, nodal* and subsequently *bmp2/4* signals define the ectoderm in the animal hemisphere, with consequent formation of oral, aboral and ciliary band ectoderm [34, 50]. *Nodal,* in turn, modulates the expression of its antagonist *lefty* and *bmp2/4*. Since *bmp2/4* is essential for the expression of all dorsal genes, its over-expression causes the formation of embryos with a dorsal part strongly developed, as confirmed by our morphological data. In our experiments, after 6 h from the fertilization of *P. lividus* in the presence of C-NPs, the mRNA levels of *nodal* and *lefty* were slightly over-expressed, explaining why *bmp2/4* was up-regulated in the presence of all C-NPs quantities. Moreover, *bmp2/4* over-expression led to the formation of strongly backbone embryos, confirmed by morphology. In vertebrates, *tbx2/3* is a target of *bmp2/4* along the dorsal–ventral patterning of the optic cup [51]. Similarly, in the *phylum* of hemichordata, phylogenetically related to the echinoderm, *tbx2/3* is also a target of *bmp2/4* suggesting that the key genes which regulate the GRN along dorsal–ventral axis are conserved [52]. Our data, showing that the over-expression of *bmp2/4* induces down-regulation of *tbx2/3,* are in agreement with literature data [25].

Another family of genes that plays a crucial role in ectoderm patterning is the *wnt* family [25]. *Wnt* signalling from the vegetal pole region is required to restrict formation of the animal pole domain, which is a small ectodermal territory characterized by the presence of thick cilia in the apical region of the embryo [53]. During *P. lividus* development, *wnt5* is a secondary target of *bmp2/4* [54]. We detected that the C-NPs treatment (medium and high C-NP quantities) strongly down-regulated *wnt5*. The downregulation of *wnt8* in response to the treatment is related to the reduction of nodal expression and suggests that the *wnt8* expression acts adversely on the autoregulation of *nodal* [55]. This observation is consistent with other studies on the GRN, which report that the direct target genes are more modulated than the indirect target genes [26, 56]. As suggested by our experiments, alterations affecting the driver gene, i.e. nodal, have a greater effect compared to modifications in downstream genes, i.e. bmp2/4, tbx2/3 and wnt5. According to data in literature, *univin* expression in early gastrula stage is limited to the ciliary band whose formation is also due to the presence of signals induced by *nodal* and *bmp2/4* [25]. On the contrary, other studies observed that the over-expression of *bmp2/4* inhibits the expression of all genes involved in ciliary band formation, including *univn,* that, in turn, causes an over-expression of all dorsal ectoderm markers [57]. The over-expression of *univin* observed in our study is due to *nodal* and *bmp2/4* increased expression, and is in line with the study of Saudemont [25].

RETRACTED ARTICLE

RETRACTED ARTICLE

Univin is also involved in the endoskeleton development in sea urchin embryos and its expression increases in parallel with the expression of *SM30* during the biopolymerization of the spicules [58]. At the vegetal pole where after the fifth division are formed micromeres, the primary mesenchyme cells (PMCs) (involved in the formation of the spicules) synthesize the specific msp130 protein and start to store SM50, SM30 and PM27 proteins [59]. SM50, SM30 and PM27 are the matrix spicules proteins and are copiously present on the spicules surface. Our data showed an increasing *SM50, SM30 and PM27* gene expression as expected since univin is a regulatory gene for skeleton genes. Indeed, *univin* is first expressed at the early blastula stage in a wider equatorial ectoderm zone. At the early gastrula stage, *univin* expression is limited to the alleged ciliary band and to the alleged arms that will develop at the pluteus larval stage [60–62]. Moreover, as already demonstrated in our previous work, the presence of C-NPs induces an alteration in the biomineralization processes that lead to the formation of embryos with short arms or spicules intertwined at the top of the pluteus larva [23].

In conclusion, genotoxicity and gene expression analyses demonstrated that NPs impaired DNA and interrupted the regular *P. lividus* development. C-NPs embryotoxicity to *P. lividus* embryos is manifested by a high incidence of asynchronous and asymmetrical divisions and endoskeleton damages. Pieces of evidence show that DNA damage and alterations in the expression of the GRN are responsible for both organism deaths and embryonic malformations. Overall, this study gives evidence that the presence of C-NPs in the marine environment is harmful to the most sensitive stages of the life cycle of the sea urchin, and confirms the E-NPs environmental risk, in particular when E-NPs are indiscriminately discharged [illegible] sea interfering with the maintenance of popul[illegible].

Supplementary Information The online version contains supplementary material available at https://doi.org/10.1007/s43188-020-00081-y.

Acknowledgements The authors would like to gratefully acknowledge Prof. D. Manno for the synthesis of C-NPs and Dr. C. Vergallo for DLS assay of C-NPs.

Author contributions CE designed, performed the experiment; TBA, MS, SA performed the experiments; DL designed the experiments, analysed the data and supervised the realization of the study; FM critically [illegible] the manuscript; CE and DL wrote the paper. Authors have read and approved the manuscript.

Funding Open access funding provided by Università degli Studi di Roma La Sapienza within the CRUI-CARE Agreement.

Compliance with ethical standards

Conflict of interest The authors declare no competing financial interest.

Open Access This article is licensed under a Creative Commons Attribution 4.0 International License, which permits use, sharing, adaptation, distribution and reproduction in any medium or format, as long as you give appropriate credit to the original author(s) and the source, provide a link to the Creative Commons licence, and indicate if changes were made. The images or other third party material in [illegible] article are included in the article's Creative Commons licence, unless indicated otherwise in a credit line to the material. If material is not included in the article's Creative Commons licence and your intended use is not permitted by statutory regulation or exceeds the permitted use, you will need to obtain permission directly from the copyright holder. To view a copy of this licence, visit http://creativecommons.org/licenses/by/4.0/.

References

1. Selck H, Handy RD, Fernandes TF, Klaine SJ, Petersen EJ (2016) Nanomaterials in the aquatic environment: a European Union-United States perspective on the status of ecotoxicity testing, res[illegible] priorities, and challenges ahead. Environ Toxicol Chem 35:1055–1[illegible]67. https://doi.org/10.1002/etc.3385
2. Bundschuh M, Filser J, Lüderwald S, McKee MS, Metreveli G, Schaumann GE, Schulz R, Wagner S (2018) Nanoparticles in the environment: where do we come from, where do we go to? Environ Sci Eur 30:6. https://doi.org/10.1186/s12302-018-0132-6
3. Prajitha N, Athira SS, Mohanan PV (2019) Bio-interactions and risks of engineered nanoparticles. Environ Res 172:98–108. https://doi.org/10.1016/j.envres.2019.02.003
4. Mahaye N, Thwala M, Cowan DA, Musee N (2017) Genotoxicity of metal based engineered nanoparticles in aquatic organisms: a review. Mutat Res 773:134–160. https://doi.org/10.1016/j.mrrev.2017.05.004
5. Gallo A, Manfra L, Boni R, Rotini A, Migliore L, Tosti E (2018) Cytotoxicity and genotoxicity of CuO nanoparticles in sea urchin spermatozoa through oxidative stress. Environ Int 118:325–333. https://doi.org/10.1016/j.envint.2018.05.034
6. Xia XR, Monteiro-Riviere NA, Mathur S, Song X, Xiao L, Oldenberg SJ, Fadeel B, Riviere JE (2011) Mapping the surface adsorption forces of nanomaterials in biological systems. ACS Nano 5:9074–9081. https://doi.org/10.1021/nn203303c
7. Mesarič T, Sepčić K, Drobne D, Makovec D, Faimali M, Morgana S, Falugi C, Gambardella C (2015) Sperm exposure to carbon-based nanomaterials causes abnormalities in early development of purple sea urchin (*Paracentrotus lividus*). Aquat Toxicol 163:158–166. https://doi.org/10.1016/j.aquatox.2015.04.012
8. Klaine SJ, Alvarez PJ, Batley GE, Fernandes TF, Handy RD, Lyon DY, Mahendra S, McLaughlin MJ, Lead JR (2008) Nanomaterials in the environment: behaviour, fate, bioavailability and effects. Environ Toxicol Chem 27:1825–1851. https://doi.org/10.1897/08-090.1
9. Gambardella C, Ferrando S, Morgana S, Gallus L, Ramoino P, Raverac S, Braminid M, Diaspro A, Faimali M, Falugi C (2015) Exposure of *Paracentrotus lividus* male gametes to engineered nanoparticles affects skeletal bio-mineralization processes and larval plasticity. Aquat Toxicol 158:181–191. https://doi.org/10.1016/j.aquatox.2014.11.014
10. Giannetto A, Cappello T, Oliva S, Parrino V, De Marco G, Fasulo S, Mauceri A, Maisano M (2018) Copper oxide nanoparticles induce the transcriptional modulation of oxidative stress-related

RETRACTED ARTICLE

genes in *Arbacia lixula* embryos. Aquat Toxicol 201:187–197. https://doi.org/10.1016/j.aquatox.2018.06.010

11. Canesi L, Ciacci C, Betti M, Fabbri R, Canonico B, Fantinati A, Marcomini A, Pojana G (2008) Immunotoxicity of carbon black nanoparticles to blue mussel hemocytes. Environ Int 34:1114–1119. https://doi.org/10.1016/j.envint.2008.04.002
12. Canesi L, Ciacci C, Vallotto D, Gallo G, Marcomini A, Pojana G (2010) In vitro effects of suspensions of selected nanoparticles (C60 fullerene, TiO_2, SiO_2) on Mytilus hemocytes. Aquat toxicol 96:151–158. https://doi.org/10.1016/j.aquatox.2009.10.017
13. Canesi L, Fabbri R, Gallo G, Vallotto D, Marcomini A, Pojana G (2010) Biomarkers in *Mytilus galloprovincialis* exposed to suspensions of selected nanoparticles (nano carbon black, C60 fullerene, nano-TiO_2, Nano-SiO_2). Aquat Toxicol 100:168–177. https://doi.org/10.1016/j.aquatox.2010.04.009
14. Nielsen HD, Berry LS, Stone V, Burridge TR, Fernandes TF (2008) Interactions between carbon black nanoparticles and the brown algae *Fucus serratus*: inhibition of fertilization and zygotic development. Nanotoxicol 2:88–97. https://doi.org/10.1080/17435390802109185
15. Radhika Rajasree SR, Ganesh Kumar V, Stanley Abraham L, Indabakandan D (2011) Studies on the toxicological effects of engineered nanoparticles in environment—a review. Int J Appl Bio Eng 5:35–45. https://doi.org/10.18000/ijabeg.10083
16. Miglietta ML, Rametta G, Francia GD, Manzo S, Rocco A, Carotenuto R, Picione LDF, Buono S (2011) Characterization of nanoparticles in seawater for toxicity assessment towards aquatic organisms. Sens Microsyst 91:425–429. https://doi.org/10.1007/978-94-007-1324-6_69
17. Carata E, Panzarini E, Dini L (2017) Environmental nanoremediation and electron microscopies. Nanotechnol Environ Remedi[illegible]t Appl Implic. https://doi.org/10.1007/978-3-319-53162-5_4
18. Carata E, Tenuzzo BA, Arnò F, Buccolieri A, Serra A, M[illegible]o D, Dini L (2012) Stress response induced by carbon nano[illegible]rticles in *Paracentrotus lividus*. Int J Mol Cell Med IJMCM 1:30[illegible]
19. Falugi C, Aluigi MG, Chiantore MC, Privitera D, Ram[illegible] P, Gatti MA, Fabrizi A, Pinsino A, Matranga V ([illegible]2) Toxicit[illegible] of metal oxide nanoparticles in immune cells o[illegible]he se[illegible]rchin. Mar Environ Res 76:114–121. https://doi.or[illegible].1016/j.[illegible]renvres.2011.10.003
20. Matranga V, Corsi I (2012) Toxic effe[illegible] of engineered nanoparticles in the marine environment: model [illegible]nisms and molecular approaches. Mar Environ Res 7[illegible]2–40. https://doi.org/10.1016/j.marenvres.2012.01.006
21. Miller RJ, Bennett S, Keller AA, [illegible]ease S, Lenihan HS (2012) TiO_2 nanoparticles are pho[illegible]oxic t[illegible] marine phytoplankton. PLoS ONE 7:e30321. htt[illegible]doi[illegible]10.1371/journal.pone.0030321
22. Ates M, Daniels [illegible] Ars[illegible] Z, Farah IO, Rivera HF (2013) Comparative evalu[illegible]ion of imp[illegible]t of Zn and ZnO nanoparticles on brine shrim[illegible] (*A[illegible]ia salina*) larvae: effects of particle size and solubility on toxici[illegible]nviron Sci Process Impacts 15:225–233. https:/[illegible]doi.org/10.10[illegible]9/c2em30540b
23. Ma[illegible] D, Cara[illegible]a E, Tenuzzo BA, Panzarini E, Buccolieri A, Filippo [illegible] Rossi M, Serra A, Dini L (2012) High ordered biomin-er[illegible]zation [illegible]nduced by carbon nanoparticles in the sea urchin *Pa[illegible]trotus lividus*. Nanotech 23:495104. https://doi.org/10.[illegible]88/0957-4484/23/49/495104
24. M[illegible]o D, Serra A, Buccolieri A, Panzarini E, Carata E, Tenuzzo B, [illegible]zzo D, Vergallo C, Rossi M, Dini L (2013) Silver and carbon nanoparticles toxicity in sea urchin *Paracentrotus lividus* embryos. BioNanoMat 14:229–238. https://doi.org/10.1515/bnm-2013-001
25. Saudemont A, Haillot E, Mekpoh F, Bessodes N, Quirin M, Lapraz F, Duboc V, Rottinger E, Range R, Oisel A, Besnardeau L, Wincker P, Lepage T (2010) Ancestral regulatory circuits governing ectoderm patterning downstream of nodal and BMP2/4 revealed by gene regulatory network analysis in an echinoderm. PLoS Genet 6:e1001259. https://doi.org/10.1371/journal.pgen.1001259
26. Su YH, Li E, Geiss GK, Longabaugh WJ, Krämer A, Davidson EH (2009) A perturbation model of the gene regulatory network for oral and aboral ectoderm specification in the sea urchin embryo. Dev Biol 329:410–421. https://doi.org/10.1016/j.ydbio.2009.02.029
27. Arizzi Novelli A, Argese E, Tagliapietra D, Be[illegible]tiol C, Volpi Ghirardini A (2002) Toxicity of tributylin and [illegible]he[illegible]yltil[illegible]to early life-stages of *Paracentrotus lividus* (Echinoderm[illegible] Ech[illegible]noidea). Environ Toxicol Chem 21:859–864. https://doi.org[illegible]0.1002/etc.5620210424
28. Arizzi Novelli A, Losso C, Falu[illegible] C, [illegible]iani [illegible]S, Kozinkova L, Lera S, Leoni T, Manzo S, M[illegible]zziotti C, Pe[illegible]grini D, Picone M, Volpi Ghirardini A (2007) Il [illegible]st di fecondazione con il riccio di mare *Paracentrotus livid[illegible]s* (L[illegible]). Bio[illegible] Mar Mediterr 14:43–47
29. Arizzi Novelli A, Losso [illegible] Volp[illegible]rardini A, Ghetti PF (2007) Saggi di tossicità co[illegible] ricc[illegible]i mare *Paracentrotus lividus*: percorso di validazio[illegible]di metodo[illegible]gie per gli ambienti di transizione attraverso una [illegible]roce[illegible]a di controllo qualità. Biol Mar Mediterr 14:100–102
30. Oliviero [illegible]M, S[illegible]iavo S, Dumontet S, Manzo S (2019) DNA damages an[illegible]ffs[illegible]quality in sea urchin *Paracentrotus lividus* sperms expo[illegible]d to ZnO nanoparticles. Sci Total Environ 651:756–7[illegible] https://do[illegible]org/10.1016/j.scitotenv.2018.09.243
31. Na[illegible]harles F, Pruski AM (2008) Improved Comet assay for the a[illegible]ssessment of UV genotoxicity in Mediterranean sea urchin eggs. [illegible]nviron Mol Mutagen 49:351–359. https://doi.org/10.1002/em.20391

[illegible] Livak KJ, Schmittgen TD (2001) Analysis of relative gene expression data using real-time quantitative PCR and the 2(−Delta Delta C(T)) method. Methods 25:402–408. https://doi.org/10.1006/meth.2001.1262

33. Minokawa T, Rast JP, Arenas-Mena C, Franco CB, Davidson EH (2004) Expression patterns of four different regulatory genes that function during sea urchin development. Gene Expr Patterns. 4:449–456. https://doi.org/10.1016/j.modgep.2004.01.009
34. Duboc V, Rottinger E, Besnardeau L, Lepage T (2004) Nodal and BMP2/4 signaling organizes the oral-aboral axis of the sea urchin embryo. Dev Cell 6:397–410. https://doi.org/10.1016/S1534-5807(04)00056-5
35. Duboc V, Lapraz F, Besnardeau L, Lepage T (2008) Lefty acts as an essential modulator of Nodal activity during sea urchin oral-aboral axis formation. Dev Biol 320:49–59. https://doi.org/10.1016/j.ydbio.2008.04.012
36. McIntyre DC, Seay NW, Croce JC, McClay DR (2013) Short-range Wnt5 signaling initiates specification of sea urchin posterior ectoderm. Development 140:4881–4889. https://doi.org/10.1242/dev.095844
37. Shashikant T, Khor JM, Ettensohn CA (2018) From genome to anatomy: The architecture and evolution of the skeletogenic gene regulatory network of sea urchins and other echinoderms. Genesis 56:e23253. https://doi.org/10.1002/dvg.23253
38. Walters C, Pool E, Somerset V (2016) Nanotoxicology: a review. https://doi.org/10.5772/64754
39. Yue Y, Li X, Sigg L, Suter MJF, Pillai S, Behra R, Schirmer K (2017) Interaction of silver nanoparticles with algae and fish cells: a side by side comparison. J Nanobiotechnol 15:16. https://doi.org/10.1186/s12951-017-0254-9
40. Wierzbicki M, Sawosz E, Grodzik M, Hotowy A, Prasek M, Jaworski S, Sawosz F, Chwalibog A (2013) Carbon nanoparticles downregulate expression of basic fibroblast growth factor in the heart during embryogenesis. Int J Nanomed 8:6. https://doi.org/10.2147/IJN.S49745

41. Samak DH, El-Sayed YS, Shaheen HM, El-Far AH, Abd El-Hack ME, Noreldin AE, El-Naggar K, Abdelnour SA, Saied EM, El-Seedi HR, Aleya L, Abdel-Daim MM (2020) Developmental toxicity of carbon nanoparticles during embryogenesis in chicken. Environ Sci Pollut Res 27:19058–19072. https://doi.org/10.1007/s11356-018-3675-6
42. Fujitani T, Ohyama K, Hirose A, Nishimura T, Nakae D, Ogata A (2012) Teratogenicity of multi-wall carbon nanotube (MWCNT) in ICR mice. J Toxicol Sci 37:81–89. https://doi.org/10.2131/jts.37.81
43. Sharifi S, Behzadi S, Laurent S, Forrest ML, Stroeve P, Mahmoudi M (2012) Toxicity of nanomaterials. Chem Soc Rev 41:2323–2343. https://doi.org/10.1039/c1cs15188f
44. Ispas C, Andreescu D, Patel A, Goia DV, Andreescu S, Wallace KN (2009) Toxicity and developmental defects of different sizes and shape nickel nanoparticles in zebrafish. Environ Sci Technol 43:6349–6356. https://doi.org/10.1021/es9010543
45. Barreto A, Luis LG, Pinto E, Almeida A, Paíga P, Santos LHMLM, Delerue-Matos C, Trindade T, Soares AMVM, Hylland K, Loureiro S, Oliveira M (2019) Genotoxicity of gold nanoparticles in the gilthead seabream (*Sparus aurata*) after single exposure and combined with the pharmaceutical gemfibrozil. Chemosphere 220:11–19. https://doi.org/10.1016/j.chemosphere.2018.12.090
46. Angerer LM, Oleksyn DW, Logan CY, McClay DR, Dale L, Angerer RC (2000) A BMP pathway regulates cell fate allocation along the sea urchin animal-vegetal embryonic axis. Dev 127:1105–1114
47. Angerer LM, Angerer RC (2003) Patterning the sea urchin embryo: gene regulatory networks, signaling pathways, and cellular interactions. Curr Top Dev Biol 53:159–198. https://doi.org/10.1016/s0070-2153(03)53005-8
48. Range R, Lapraz F, Quirin M, Marro S, Besnardeau L, Lepage T (2007) Cis-regulatory analysis of nodal and maternal control of dorsal-ventral axis formation by Univin, a TGF-beta [illegible] to Vg1. Dev 134:3649–3664. https://doi.org/10.1242/dev.00[illegible]
49. Angerer RC, Angerer LM (2012) Sea urchin em[illegible] specification of cell fates. https://doi.org/10.1002/97804700159[illegible]0001513.pub3
50. Yaguchi S, Yaguchi J, Burke RD (2007) Sp-Smad2/3 mediates patterning of neurogenic ectoderm b[illegible] nodal in the sea urchin embryo. Dev Biol 302:494–503. https://[illegible]org/10.1016/j.ydbio.2006.10.010
51. Behesti H, Holt JK, Sowden JC ([illegible]) [illegible] level of BMP4 signaling is critical for the regulation of distinct T-box gene expression domains and growth along [illegible] dorso-ventral axis of the optic cup. BMC Dev Biol 15:6[illegible] https[illegible]org/10.1186/1471-213X-6-62
52. Lowe CJ, Terasak[illegible] M, [illegible] M, Freeman RM Jr, Runft L, Kwan K, Haigo S, Aron[illegible]wicz J, La[illegible] E, Gruber C, Smith M, Kirschner M, Gerhart J (2006) Dorsoventral patterning in hemichordates: insights into early chordate evolution. PLoS Biol 4:e291. https://doi.org/10.1371/journal.pbio.0040291
53. Ferkowicz MJ, Raff RA (2001) Wnt gene expression in sea urchin development: heterochronies associated with the evolution of developmental mode. Evol Dev 3:24–33. https://doi.org/10.1046/j.1525-142x.2001.00084.x
54. Branford WW, Yost HJ (2002) Lefty-dependent inhibition of nodal- and Wnt responsive organizer gene expression is essential for normal gastrulation. Curr Biol 12:2136–2[illegible]. [illegible]tps://doi.org/10.1016/s0960-9822(02)01360-x
55. Wei Z, Range R, Angerer R, Angerer L (2012) Ax[illegible] patterning interactions in the sea urchin embr[illegible] suppression of nodal by Wnt1 signaling. Dev 139:1662–16[illegible]9. htt[illegible]/doi.org/10.1242/dev.075051
56. Yi H, Wang Z, Li X, Yin M, [illegible]ang L, Aldalbahi A, El-Sayed NN, Wang H, Chen N, Fan C, Song [illegible] (2016) Silica nanoparticles target a wnt signal transdu[illegible] for deg[illegible]ation and impair embryonic development in zeb[illegible]sh. T[illegible]nostics 6(11):1810–1820. https://doi.org/10.7150/t[illegible].16127
57. Lapraz F, Besn[illegible]rdea[illegible] Lepage T (2009) Patterning of the dorsal-ventral axis [illegible] echinod[illegible]ns: insights into the evolution of the BMP-chordin [illegible]gnaling network. PLoS Biol 7:1–26. https://doi.org/10.[illegible]/j[illegible]bio.1000248
58. Zito F, Cos[illegible]C, Sciarrino S, Poma V, Russo R, Angerer LM, M[illegible]tranga V (2[illegible]3) Expression of univin, a TGF-beta growth factor, [illegible] ectoderm–ECM interaction and promotes skeletal grow[illegible] in the sea urchin embryo. Dev Biol 264:217–227. https://doi.org/10.1016/j.ydbio.2003.07.015
59. Wilt FH (2002) Biomineralization of the spicules of sea urchin embryos. Zool Sci 19:253–261. https://doi.org/10.2108/zsj.19.253
60. Röttinger E, Saudemont A, Duboc V, Besnardeau L, McClay D, Lepage T (2008) FGF signals guide migration of mesenchymal cells, control skeletal morphogenesis [corrected] and regulate gastrulation during sea urchin development. Dev 135:353–365. https://doi.org/10.1242/dev.014282
61. Röttinger E, Saudemont A, Duboc V, Besnardeau L, McClay D, Lepage T, Saudemont A, Haillot E, Mekpoh F, Bessodes N, Quirin M, Lapraz F, Duboc V, Röttinger E, Range R, Oisel A, Besnardeau L, Wincker P, Lepage T (2010) Ancestral regulatory circuits governing ectoderm patterning downstream of nodal and BMP2/4 revealed by gene regulatory network analysis in an echinoderm. PLoS Genet 6:1–31. https://doi.org/10.1371/journal.pgen.1001259
62. Stenzel P, Angerer LM, Smith BJ, Angerer RC, Vale WW (1994) The univin gene encodes a member of the transforming growth factor-beta superfamily with restricted expression in the sea urchin embryo. Dev Biol 166:149–158. https://doi.org/10.1006/dbio.1994.1303

RETRACTED ARTICLE
